# How Twitter Is Studied in the Medical Professions: A Classification of Twitter Papers Indexed in PubMed

**DOI:** 10.2196/med20.2269

**Published:** 2013-07-18

**Authors:** Shirley Ann Williams, Melissa Terras, Claire Warwick

**Affiliations:** ^1^School of Systems EngineeringUniversity of ReadingReadingUnited Kingdom; ^2^Department of Information StudiesUniversity College LondonLondonUnited Kingdom

**Keywords:** Twitter messaging, Twitter messenging, information science, Twitter, microblogging, papers, classification, social network systems

## Abstract

**Background:**

Since their inception, Twitter and related microblogging systems have provided a rich source of information for researchers and have attracted interest in their affordances and use. Since 2009 PubMed has included 123 journal articles on medicine and Twitter, but no overview exists as to how the field uses Twitter in research.

**Objective:**

This paper aims to identify published work relating to Twitter within the fields indexed by PubMed, and then to classify it. This classification will provide a framework in which future researchers will be able to position their work, and to provide an understanding of the current reach of research using Twitter in medical disciplines.

**Methods:**

Papers on Twitter and related topics were identified and reviewed. The papers were then qualitatively classified based on the paper’s title and abstract to determine their focus. The work that was Twitter focused was studied in detail to determine what data, if any, it was based on, and from this a categorization of the data set size used in the studies was developed. Using open coded content analysis additional important categories were also identified, relating to the primary methodology, domain, and aspect.

**Results:**

As of 2012, PubMed comprises more than 21 million citations from biomedical literature, and from these a corpus of 134 potentially Twitter related papers were identified, eleven of which were subsequently found not to be relevant. There were no papers prior to 2009 relating to microblogging, a term first used in 2006. Of the remaining 123 papers which mentioned Twitter, thirty were focused on Twitter (the others referring to it tangentially). The early Twitter focused papers introduced the topic and highlighted the potential, not carrying out any form of data analysis. The majority of published papers used analytic techniques to sort through thousands, if not millions, of individual tweets, often depending on automated tools to do so. Our analysis demonstrates that researchers are starting to use knowledge discovery methods and data mining techniques to understand vast quantities of tweets: the study of Twitter is becoming quantitative research.

**Conclusions:**

This work is to the best of our knowledge the first overview study of medical related research based on Twitter and related microblogging. We have used 5 dimensions to categorize published medical related research on Twitter. This classification provides a framework within which researchers studying development and use of Twitter within medical related research, and those undertaking comparative studies of research, relating to Twitter in the area of medicine and beyond, can position and ground their work.

## Introduction

Since their inception in 2006, Twitter and similar microblogging systems have provided data for research, with the first academic paper on the subject appearing in 2007 [1]. Articles in the popular news media highlight the potential of Twitter based research to meet a number of goals ranging from measuring public sentiment to spotting flu outbreaks [2]. However, there has been little work done beyond the headlines in understanding how or why people are using information gathered from Twitter systems for research, particularly around specific topic areas.

The terms microblog and Twitter are both widely used by authors, dating from the first paper on the subject [1]. The term microblogging is defined as:

A variant of blogging which allows users to quickly post short updates, providing an innovative communication method that can be seen as a hybrid of blogging, instant messaging, social networking and status notifications. The word’s origin suggests that it shares the majority of elements with blogging, therefore it can potentially be described using blogging’s three key concepts: the contents are short postings, these postings are kept together by a common content author who controls publication, and individual blog entries can be easily aggregated together.

3,4

Some writers hyphenate the term as “micro-blog” [5], while other do not [6]. We follow the majority and use the unhyphenated version, although while searching for papers on the topic we utilized both. Twitter is usually defined in terms as microblogging:

Twitter is a microblogging site, originally developed for mobile phones, designed to let people post short, 140-character text updates or “tweets” to a network of others. Twitter prompts users to answer the question “What are you doing?”, creating a constantly- updated timeline, or stream, of short messages that range from humor and musings on life to links and breaking news. Twitter has a directed friendship model: participants choose Twitter accounts to “follow” in their stream, and they each have their own group of “followers”.

7

PubMed is a free Web literature search service developed and maintained by the National Center for Biotechnology Information (NCBI) [8]. Since 1996, PubMed gives access to citation and abstracts of some 5400 biomedical journals covering the fields of medicine, nursing, dentistry, veterinary medicine, health care systems, and preclinical sciences. The intended users of PubMed are researchers, health care professionals, and the general public. For the intended users, PubMed serves as the primary tool for electronically searching and retrieving biomedical literature [9]. Fink [10] describes PubMed as “the best site for published medical and health research”. PubMed uses the Medical Subject Headings (MeSH) controlled vocabulary to supplement searches. MeSH pre-dates PubMed with its origins in the 1960s as a set of catalog headings across medicine composed by the US National Library of Medicine [11]. Entries to MeSH are regularly updated to match changes in medicine and technology.

In common with many other papers, we used the term Twitter to encompass all microblogging systems. The work was not a traditional literature review [10]. Instead, only papers indexed by PubMed were considered and only those related to Twitter were reviewed then classified.

This work will provide a framework with which researchers studying Twitter related topics and their applications in medical related areas will be able to position and ground their work. It will provide a single point where current work on the medical use of Twitter can be compared and contrasted. Additionally it will help to understand the scope and reach of using PubMed as a data source.

Our analysis shows that Twitter related research can be classified in a variety of ways: whether it is Twitter-focused or part of a wider social media related study; whether it is based on data, and if so, the quantity of data considered; the domain in which the work is based; the methods used; and the aspect–or characteristic–of Twitter considered. These dimensions of classification provide a framework in which Twitter-related medical research can be positioned and compared with other work within the area and beyond.

## Methods

### Data Collection

Researchers normally identify papers on a topic in a number of different ways such as chaining from existing papers and database searches [12,13]. There are many databases and search engines available to researchers wanting to find papers on a particular topic [10], some of which are freely available, while others are available via individual or institutional subscription [14]. Researchers in areas of emerging technologies sometimes limit themselves to groups of publications [15], single journal sources [16], or concentrate around conferences [17]. While many studies do not indicate their identification method, Cormode et al [18], for example, classify Twitter papers providing examples of “first studies” and the “next set of papers”. Within this work we wanted to investigate the area of Twitter based research in medicine, and for our data collection to be replicable we chose to make a structured search of journal articles.

Initial experimentation showed that for Google Scholar [19] the searches either had to be limited to searching the article’s title or it is full text. Searches limited to articles title would not return “OMG U got flu? Analysis of shared health messages for bio-surveillance” [5] as it does not contain any words related to Twitter. Full text searches returned articles which had “share this on Twitter” buttons on the page even though the article was nothing to do with microblogging. Using our institutional library’s facility to search freely available electronic resources for papers relating to Twitter in the biomedical field, we established that PubMed returned over 100 items while BioMed Central [20] returned around 20, and other databases returned very few papers, and almost all were already in the PubMed list.

Gold et al [21] faced a similar challenge when undertaking a systematic examination of the use of social networking sites for health promotion: from a systematic search of a range of databases they originally found 204 academic papers but closer investigation showed only one was relevant, a Web search revealed over 80 million electronic resources and an unknown number of social networking sites. Likewise Guse et al [22] investigated the use of digital media to improve adolescent sexual health searched a range of databases to identify 942 possible abstracts of which 10 met the inclusion criteria: while they do not indicate which databases they found each paper in, all the 10 studies can be found via PubMed.

It was determined for this study that a structured search using PubMed would be used to identify papers in journals. While this most certainly would not give an exhaustive list of papers on Twitter it does mean that the search is repeatable, by other researchers, allowing future studies to include papers added to PubMed. Using subscription based services (such as Scopus) would mean only some researchers could repeat the study limiting its usefulness as a benchmark.

The data collection was made for the papers that were first published between 2007 (the first year academic papers on Twitter appeared) and 2011 (the last complete year before this study); inclusive of papers available online as preprints ahead of the print version (epubs).

During 2010, the terms “Twitter messenging” and “Twitter messaging” were introduced into the MeSH controlled vocabulary under the headings Internet and Blogging respectively. There are no entries relating to the term microblog or its variants, although blogging is present. There are currently no papers within PubMed that are returned by searches on the MeSH terms: “Twitter messenging” or “Twitter messaging”. It should be noted that where papers have keywords, not indexed by MeSH terms, PubMed does not store these and so it is not possible to search PubMed for papers with keywords such as “Twitter” or “microblog”. Therefore, the terms Twitter, Tweet, Microblog, and Micro-blog were used as the basis for keyword searching across all fields in PubMed, and then cross-referenced and checked to remove spurious data. A total of 139 papers were initially identified which had used terms from the query in a medical context. Five of these were subsequently found to be only included in the results because one of the author’s surnames or usernames included “tweet”, and so a base corpus of 134 papers was created.

### Data Classification

Previous research [23] showed that a number of dimensions could be identified and studied when Twitter-related academic papers and their abstracts are analyzed. These include:

1. *Focus*. Papers can be predominantly about Twitter or related microblogging such as the use of the Chinese microblog site Sina Weibo [24], or they can be partially about Twitter but predominantly about other things, for example considering a number of different social networking sites of which Twitter is just one [25]. There are also unknowns where a paper has no abstract. Additionally there are papers where the term twitter is used with its conventional meaning such as a noise made by birds.
2. *Data*. The data used in studies is varied, ranging from observations of small samples, through questionnaires, to collecting vast quantities of information via the Twitter API (an interface that allows technically skilled users to extract data). The date of the study also impacts on the timeliness, quantity and quality of data.
3. *Domain*. Studies are undertaken from a number of different standpoints and often within a domain or a group of domains.
4. *Method*. Researchers use a variety of methodological techniques when carrying out research into Twitter.
5. *Aspect*. The aspect or characteristic of Twitter considered. Many studies concentrate on looking at the message (tweets), while others study the user (tweeter), with smaller numbers look at the underlying technology and how it can be developed. A number of papers consider the concept of Twitter without any detail of its use.

The overarching approach to classification was based on the approach used in a study of research on microblogging in education [15], with independent coding and then discussion until consensus was reached. For each paper in our corpus, the focus was identified, based on close reading of the title and abstract. Those papers identified as Twitter-focused were subject to a qualitative classification on the title, abstract and full paper using open coded analysis to determine groupings for the data used in the work described. Corbin and Strauss [26] have shown how this methodology facilitates the breaking of corpora data into delineated concepts as well as featuring in grounded theory [27] where initial and focused line by line coding produces label variables from within the data itself. The approach has been previously used successfully to classify Twitter posts [4]. The grouping of method, domain and aspect was initially identified from the paper’s title and abstract and verified by consulting the full paper.

## Results

### Focus

Multimedia Appendix 1 summarizes the flow of selection of papers from our base corpus of 134 papers. From this corpus thirty [5,6,28-55] were Twitter-focused. The papers had a significant proportion that was related to some aspect of microblogging. For example Chew and Eysenbach [31] in their paper entitled “Pandemics in the age of Twitter: content analysis of Tweets during the 2009 H1N1 outbreak” study how Twitter was used in relation to the spread of infection in a pandemic.

There were 57 corpora [21,56-111] that mentioned Twitter but were primarily about another topic. For example Turner-McGrievy and Tate [105] in their paper, “Tweets, Apps, and Pods: Results of the 6-month Mobile Pounds Off Digitally (Mobile POD) randomized weight-loss intervention among adults” study a combination of podcasts and other techniques including using Twitter in relation to weight loss.

Out of 134 papers, 36 [112-147] had no abstract, for example the article “Are you using Twitter for your next survey?” by Pattillo [127]. Further investigation showed that this is a news article within the publication. Papers without abstracts are therefore not considered in any further detail, given that they were news reports rather than academic articles per se. News stories have been shown to be rated differently by medical professionals according to their authorship [148]. Wilson et al [149] took a similar decision to concentrate on academic papers when reviewing papers related to Facebook, and highlighting that while unpublished manuscripts, dissertations, position papers, and popular press articles offer thoughtful insights, their quality is variable.

Out of 134 articles, there were 11 [150-160] not related to microblogging, with 10 of these the term “twitter” being used with original, non-microblogging meanings. For example “Why do shrews twitter? Communication or simple echo-based orientation” [156] is about the noise made by shrews. Exceptional was a paper entitled “Plant twitter: ligands under 140 amino acids enforcing stomatal patterning” [159], as the paper is not about microblogging but in the area of plant research. The MeSH terms used to classify the paper support this, but interestingly the only appearance of “twitter” is in the title; a form of pun. These non-microblogging papers are not considered in any further detail.

Table 1 shows the number of Twitter-focused papers and the number of papers mentioning Twitter published each year between 2007 and 2011, and compares them with the numbers for general journals [23], found by searching Scopus [161] and Web of Science within Web of Knowledge [162]. Note there were no such papers published in medical fields in 2007 and 2008, although they were appearing in other disciplines. Since 2009 the number of papers has increased each year. This analysis suggests that although the use of Twitter in medical research came later than in some other disciplines, its use is growing and its importance is increasing as time progresses. Initial indications for 2012 suggest that the number of papers published both in the area of medicine and more generally will be greater than the numbers published in 2011.

The 2 papers in the corpus published in 2009 [28,29] and 3/8 published in 2010 [30,32,36] discussed the merits of Twitter and whether it should be used by medical professionals. The study of Twitter content for medical related terms was first seen within the corpus in 2010 papers [31,35], while general examination of terms was first presented in 2007 [1].

In the following we consider only the Twitter-focused papers in medical related disciplines. Those papers that use Twitter or other microblogs as a primary source and topic for research as identified via PubMed. Multimedia Appendix 2 combines the information presented in Tables 1-5 for all the Twitter-focused papers.

### Data

Across the papers a number of different types of data sources were reported including surveys, user profiles, tweets (posts), and individual words in tweets. The size of data set examined ranged from small, with a few items, to large scale, with billions of individual data points. Some papers were not based on data, particularly those early papers that were introducing the concept of Twitter.

For some papers the abstracts indicated the data studied, for example in a paper “Use of Twitter to encourage interaction in a multi-campus pharmacy management course” [41] the abstract includes the following:

More than eighteen hundred tweets were made by students, guests, and the instructor... One hundred thirty-one students completed an optional evaluation survey.

41

Indicating the type of data and quantities, the full paper shows that the students posted 1775 tweets over 6 days, as well as indicating the use by other participants. The Twitter data was collected by graduate teaching assistants using a Twitter list in preference to hashtags, which the students are reported to have found cumbersome. In other papers, the abstract provides only partial information about the dataset. For example in a paper “Social media & stem cell science: examining the discourse” [38], the abstract indicates that Twitter posts are analyzed. But the full paper needs to be consulted to identify that the researchers used TweetDeck to collect 2 sets of tweets, one group of 35 using the term “DeGette” over a 6 day period, and a group of 50 using “trachea stem cells” over a 4 day period. Similarly, the paper “Diurnal and seasonal mood vary with work, sleep, and day length across diverse cultures” [43] indicates in the abstract that millions of Twitter messages are considered, the full paper provides more details:

Using Twitter.com’s data access protocol, we collected up to 400 public messages from each user in the sample, excluding users with fewer than 25 messages. The resulting corpus contained about 2.4 million individuals from across the globe and 509 million messages authored between February 2008 and January 2010.

43

The paper “Implementing Twitter in a health sciences library” [32] is a report on the establishing of a Twitter presence by the communications team within the library. The work is not based on data although in the evaluation section the authors do report on the number of followers (66) the account has gathered and classifying these in relationship to the library.

Stratifying across the different descriptions of data we identified 4 categories which can be used to describe the datasets used to study Twitter in a medical context.

1. *Large*. Studies looking at vast amounts of data that would require a team of researchers and the use of automated tools if the data is to be analyzed in a timely manner. Typically considering over a million tweets and/or a million accounts. The term “big data” is often used to describe the quantity of data in such studies
2. *Medium*. Studies using quantities of data that could realistically be analyzed manually by a dedicated researcher or a small team with limited tool support. Typically considering thousands of tweets or accounts.
3. *Small*. The data handled could be reasonably handled by a researcher alongside other tasks. Typically considering surveys, groups, tweets, and user profiles, with up to a thousand items.
4. *Not data based*. Papers not based on data collection and analysis.

Table 2 shows the categorization of data in the Twitter related papers by year published. The early papers (2009 and 2010) were predominantly not based on data, typically explaining the affordances of Twitter. In 2011 all papers had a data element, while there were a range of papers using large, medium, and small scale datasets. There is an increase in large scale analysis of Twitter from 1 study in 2010 to 6 in 2011, indicating that computational analysis of large scale datasets of Twitter data are becoming more common.

### Domain

All the papers in this study are from PubMed and so the broad domain is medical, however the researchers have a number of different standpoints. Consideration was given to the selection of domains from sub-area and disciplines of medicines, but typically there are only a few papers in each sub-area, see Table 3.

Based on an analysis of the contents of full papers we have identified the following broader topic, or domain, areas. Some papers are allocated to more than one of these domains:

- *Academic*. Seven papers in total [30,32,34,37,40,41,48] have an academic perspective ranging through education for professions, libraries, and scholarly publications, to an experimental use of Twitter with groups of students.
- *General Communication*. Fourteen papers [5,6,31,35,39,43-46,50-54] examine the general Twitter interface, and do not in any ways select individuals. These include all the papers which analyze large scale datasets.
- *Medical Professional Communication*. Nine papers [32,33,36,38,40,42,47,48,55] consider use by professionals within an area, both among themselves and with patients, as well as one way communication to the more general public (including marketing).
- *Targeted Communication*. Two papers [38,49] involve other identifiable groups not related to medical professionals. one was an analysis of accounts that were identified as related to quitting smoking [49].
- *Guides*. Four of the papers [28-30,36] are written primarily as guides: all of these concentrated on explaining the concept and purpose of Twitter.

**Table 1:** Number of Twitter related papers published per year.

Year	Mentions Twitter (Medical)	Twitter-focused (Medical)	Mentions Twitter (General)	Twitter-focused (General)
2007	0	0	3	3
2008	0	0	12	8
2009	6	2	70	36
2010	18	8	217	210
2011	33	20	248	320

**Table 2:** Data categorization of Twitter papers by year.

Year	Large	Medium	Small	Not data based
2009				2
2010	1	1	2	4
2011	6	7	7	

**Table 3:** Sub-areas and number of papers.

Sub-area	Number of papers
Psychology	5
General	4
Influenza	3
Neurology	3
Pharmacy	3
Administration	2
Happiness	2
Nursing	2
Dentistry	1
Health education	1
Information science	1
Natural science	1
Orthopaedics	1
Sociology	1

### Methods and Aspects

Initially, the papers’ titles and abstracts were read to try to identify the methodological approach use by the researchers. For the papers with structured abstracts and some others this clearly indicated the approach taken. For example a paper entitled “'What's happening?' A content analysis of concussion-related traffic on Twitter” [54] clearly used a content analysis approach. Following this initial pass, all papers were examined for details of methods used. An open coding approach was used to capture the diversity of approaches. This resulted in across the 30 papers 53 methods identified, and not all of which were distinct, see Table 4.

These methods were then stratified into 3 broad categories:

1. *Analytic*. Where the researchers had performed some type of analysis, which may be quantitative or qualitative. Sometimes these methods are supported by existing or new techniques from artificial intelligence, mathematics and statistics to facilitate knowledge discovery and mining of information. Many of the papers use the techniques of content analysis: for example in “Pandemics in the age of Twitter: content analysis of Tweets during the 2009 H1N1 outbreak” [31], while in “OMG U got flu? Analysis of shared health messages for bio-surveillance” [5] machine learning techniques are used alongside content analysis. Social network analysis is used in the paper “Modeling users' activity on twitter networks: validation of Dunbar's number” [44] to extract and analyze 25 million conversations from some 380 million tweets.
2. *Design and Development*. Where systems are proposed or built, to interact with Twitter, such systems are often demonstrators used by the authors within their own context. For example, in a paper entitled, “A new support system using a mobile device (smartphone) for diagnostic image display and treatment of stroke” [55], the method of the work is presented as the creation of a communication system that was piloted in the author’s hospital, the system includes the capability to tweet to other professionals. While in “Machine intelligence for health information: capturing concepts and trends in social media via query expansion” [52], the authors develop information retrieval techniques to facilitate working with their Twitter corpus, and in “A visual backchannel for large-scale events” [33] they describe a system they have developed and trials that allows the tweets related to an event to be presented graphically.s
3. *Examination*. Where the authors had undertaken review and survey type works, including approaches such as: case studies, categorizations, essays, ethnographic studies, interviews, and investigation. For example in a paper entitled, “Twitter as a communication tool for orthopedic surgery” [42], they identified, categorized, and reviewed Twitter profiles of over 400 orthopedic professionals. While in a paper entitled “Should you be tweeting?” [28], interviews with scientists who use Twitter are presented. This paper would itself be classed as an examination paper.

Alongside the methods the aspect of Twitter primarily considered in research was identified according to the 4 categories:

1. The messages (tweets).
2. The users (tweeter).
3. The underlying technology and how it can be developed.
4. The concept of Twitter without any detail of its use.

For all medical related papers it was possible to identify a primary method and primary aspect considered by the researchers and these are summarized in Table 5. Some papers also were identified as having secondary aspects, as shown in Multimedia Appendix 2.

It is interesting to note that the majority of the papers report research using analytic methods, and the majority of this group look at the contents of the tweets sent, rather than the users. The 6 papers using examination methods such as reviews considering the concept of Twitter are the same as the 6 papers in Table 2 that are not based on data. A similar classification of general papers [23] identified proportionally many more papers using the design and development methods. The general papers 154 of the total 575 papers primarily using a design and development method on the message aspect. None of the PubMed papers took this approach. Otherwise the PubMed papers do have a similar spread to the general papers.

**Table 4:** Methodological approaches initially identified.

Methods identified	Number of papers
Content analysis	12
Review	4
Survey	4
Experimental	2
Graph	2
Machine intelligence	2
Mined	2
Statistical	2
System development	2
System implementation	2
Algorithmic	1
Analysis	1
Automation	1
Classification	1
Classification analysis	1
Comparative analysis	1
Correlation analysis	1
Evaluation	1
Examination	1
Investigation	1
Mathematical	1
Model	1
Normalisation	1
Qualitative	1
Simulation	1
Statistics	1
System design	1
Text analysis	1
Text mining	1

**Table 5:** Number of papers with primary method and aspect.

	Message	User	Technology	Concept	Total
Analytic	11	5	0	0	16
Design and development	0	0	4	2	6
Examination	1	1	0	6	8
Total	12	6	4	8	

## Discussion

### Principal Results

Across PubMed 123 papers were identified that were Twitter related; this is a very tiny proportion of the more than 21 million citations held in the database. The first papers indexed by PubMed were published in 2009, 3 years after the launch of Twitter and 2 years after the first Twitter papers appeared in other disciplines. The early Twitter focused papers introduced the topic and highlighted the potential, not carrying out any form of data analysis. However subsequent studies analyzed quantities of Twitter data and one of the principal findings of this study is the size of studies that are now possible based on Twitter in the medical field. The first of the large studies of over a million pieces of data was published in November 2010 [31]. Researchers are now reporting collecting billions of items of data over almost 3 years [6]. Collecting large quantities of data is challenging, as explained,

Our research material of tweets was gathered by using the Twitter4J … an open-source Java library for the Twitter Application Programming Interface (API). The tweets were stored locally as Twitter limits online search to one week. This strategy allowed an increased sample size improving the likelihood of detecting trends. Twitter API provided approximately one per cent of all real-time tweets. Our tweet corpus included English tweets over fourteen days. The data was gathered during 4 Jan 2011 at 13:36–20:10 EST with 300,000 tweets and 582,975 words.

52

The Edinburgh Twitter corpus of 97 million tweets was used in one paper [5], however that corpus is no longer available due changes to Twitter’s current terms and conditions [163]. This means researchers are no longer able to share corpuses of Twitter data and so the handling of large sets of data need teams to include the expertise and capacity to extract, store and manipulate large quantities of information. Teams also need to be aware of limitations placed by Twitter on developer’s access to Twitter data and the possibilities of changes during the lifetime of a project. Likewise the methods for understanding the data collected are moving on from what can be undertaken by lone researchers using qualitative approaches, and while the methods used are still broadly analytic they are using techniques from knowledge discovery and mining of information [40].

### Limitations

Limiting the papers examined in this study to those indexed in PubMed between 2007 and 2011 means that there is a body of work published since the start of 2012 that is not considered. While PubMed indexes some 5400 journals there are journals not indexed, including those not in English. A lot of papers published on the subject of Twitter are in conference proceedings. For instance, the Scopus database [161] returns approximately twice as many conference papers as journal papers on the subject (across all fields not just medicine), and there are many conferences that are not indexed. Over and above papers there are many blog posts reporting medical use of Twitter. For example, Bottles [164] describes his personal use of Twitter, and Neylon [165] discusses links shared by nurses. However there is no reliable way of identifying all such posts, nor is it possible to guarantee the posts will remain available. The selection of a single data source does mean that the study is reproducible, and based on published, peer-reviewed research rather than accounts and reflections by individuals. Future comparison can be done on a year by year basis to trace the changing use of Twitter in the medical domain.

Searching on the MeSH terms did not prove useful in highlighting relevant papers. Given the terms “Twitter messaging” and Twitter messenging” were only added to the vocabulary during 2010 this is not totally surprising, although we did expect to see some use of these terms in the most recent publications. This indicates that the MeSH vocabulary system is not being adequately used by authors and publications writing about Twitter, which is problematic given that it is the only faceted search available in PubMed.

The word “twitter” is sometimes used in medical related research with its original meaning. Papers that did this were discounted from this study. Potentially papers may be incorrectly excluded, for example a paper that related both patients with twitters and who used microblogging. We do not believe this was the case in the papers considered here but it is certainly a potential limitation with the approach.

Given that this paper covers only the first few years of academic research in the area of Twitter, it is likely that some of the approaches reported upon are fledgling and that over the next years the methods applied will reach a degree of maturity that will impact on the broad methodological classification presented here.

### Analysis of Papers’ Findings

The papers reviewed and categorized here were diverse in their finding and conclusions. Of the findings many were closely linked to the domain of study rather than the use of Twitter or social media in general. For example, the findings and conclusions of Golder and Macy [43] all relate to mood change and day patterns. There was no discussion as to the use of Twitter as a source of data.

In the papers in the domain of professional communications, where usually papers concentrate on the concept of Twitter, rather than findings extrapolated from Twitter data, the approach was usually a review or other method classified above as examination. These tended to conclude that they had introduced Twitter and highlighted its potential. Although some were less enthusiastic.

Despite the growing popularity of social media across multiple disciplines, the majority of pharmacy preceptors surveyed were not willing to use these venues in professional practice.

47

Papers looking at medium and large data sets often included indications that their work illustrated the potential for studies in medical related area to use Twitter and other social media data.

The study adds to evidence supporting a high degree of correlation between pre-diagnostic social media signals and diagnostic influenza case data, pointing the way towards low cost sensor networks.

5

Also among these studies authors indicate that the abundance of data will change the way in which researchers approach their studies [6].

### Conclusions

This work is to the best of our knowledge the first broad study of medical related research based on Twitter and related microblogging. We have identified that medical related research in this area was first published in 2009 and that the number of papers has increased in both the following years.

From the some 5400 journals indexed by PubMed, we have identified thirty papers that focus on Twitter and 57 that mention it. There are also a number of papers in which the term twitter is used with its original meaning and not at all related to microblogging. There are some papers indexed that appear to relate to Twitter but do not have abstracts further investigations shows these to be editorial or news type items as opposed to academic oriented papers. Further work will need to be undertaken to identify and classify work beyond the academic papers indexed by PubMed, this would include diverse sources such as book chapters, conference proceedings, and blog posts.

While the early Twitter-focused papers were predominantly introductory explaining to the readership what Twitter was about and considering its potential, we are now seeing work reported were researchers have examined large quantities of Twitter data, using these large data sets to obtain better understanding of topics within medicine. We have classified this usage of data into 4 categories: large, medium, small, and no data. This access to large amount of data stemming from individual tweets coupled with metadata of location, time of day, networks of followers holds potential for many future studies building on existing work such as identification of the spread of infectious diseases but it has also potential for the identification of previously impossible studies based on personal thoughts put into a public space. While most studies use methods that can be broadly classed as analytic, the large quantities of data mean that analysis techniques that facilitate knowledge discovery and mining of information are starting to be used. As the number of research papers grows, the dimension of domain will need to be revisited as other stratifications may become possible.

The results presented here will provide researchers with an insight into the medical domain and Twitter use, where there is work in related sub-areas that can be used to inform new studies and those that have still to be studied rigorously. The large data studies that have completed certainly have information on techniques for data collection and method for analysis that will be useful in other domains. Identifying areas where further research is needed is difficult, but we would suggest that the following are neglected areas within the realms of twitter and medicine:

- *Outreach and investigating the reach and scope of Twitter messages.* Although Prochaska et al [49] have reviewed the content of accounts related to Quitting Smoking, none of the studies have investigated the reach of such accounts, or the best ways to use them.
- *Public engagement*. While Adams et al [38] have investigated what is said about their subjects, there are no investigations where discussion is invited or prompted surrounding medical areas.
- *Legal and ethical issues*. While a number of papers (particularly the early ones [28,29]) discuss the general use there are no academic studies of the ethical issues of medical professionals using Twitter, nor any detailed studies of the legal implications of using Twitter in a medical context.

This study provides a framework within which researchers studying the development and use of Twitter within medical related research will be able to position their work and against those undertaking comparative studies of research relating to Twitter in the area of medicine and beyond will be able to ground their work. We have provided an analysis of the use and usefulness of microblogging within medical fields at a time when social media is being increasingly used for research purposes across many domain and in a reproducible manner, which can be built upon in future as more studies are published.

## Multimedia Appendix 1

Flow diagram of search strategy.

## Multimedia Appendix 2

Overview table.

## Abbreviations

NCBI: National Center for Biotechnology Information
MeSH: Medical Subject Headings
