# Supplementary figures and images for: How Twitter Is Studied in the Medical Professions: A Classification of Twitter Papers Indexed in PubMed
Source: Med 2 0. 2013 Jul 18;2(2):e2. doi: 10.2196/med20.2269 (PMC4084770; doi:10.2196/med20.2269)

## ***Appendix A - Flow diagram of search strategy***

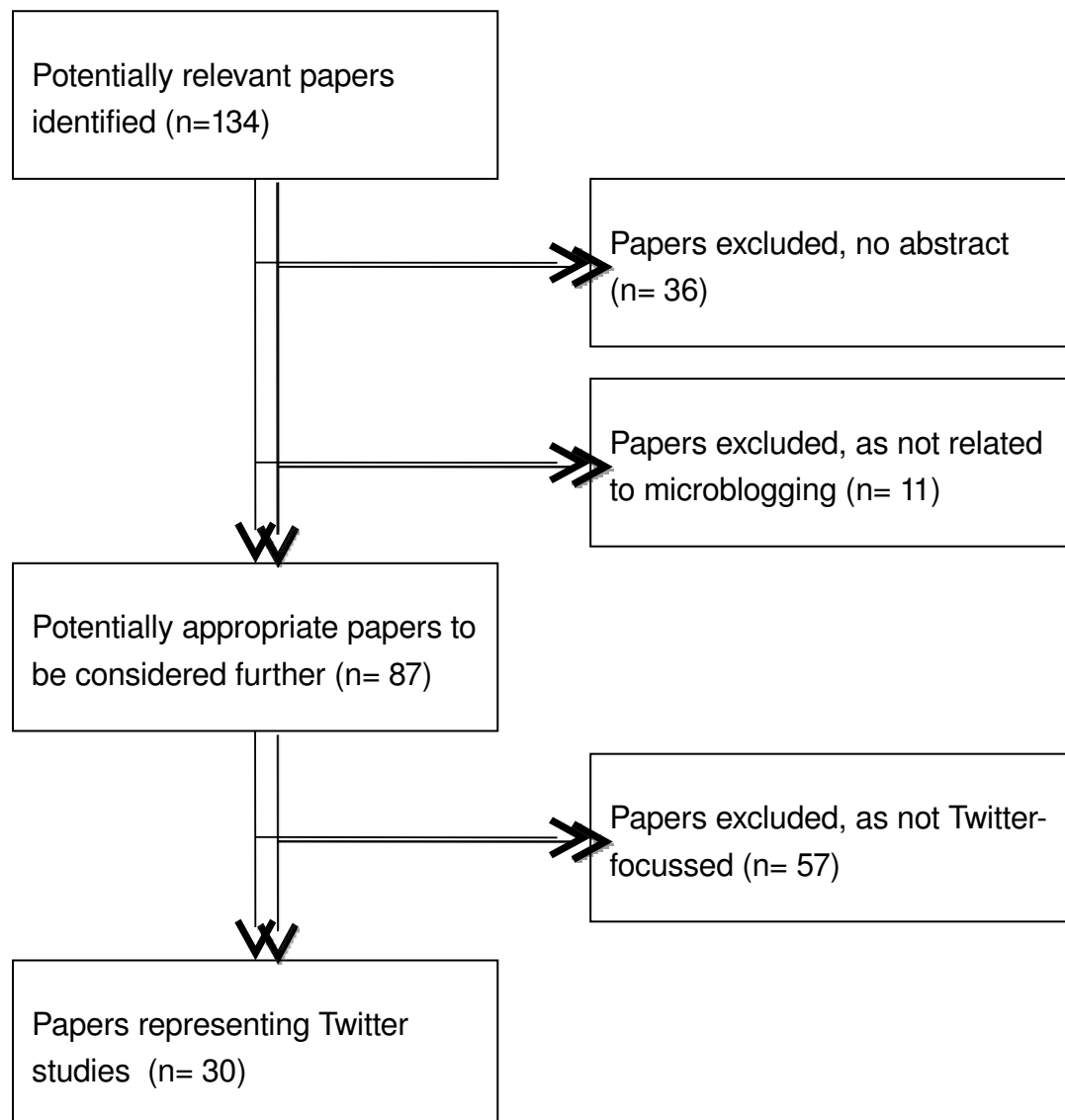

Supplement: Supplementary file 1 [file med20_v2i2e2_app1.pdf]
