# Supplementary material for: How Twitter Is Studied in the Medical Professions: A Classification of Twitter Papers Indexed in PubMed
Source: Med 2 0. 2013 Jul 18;2(2):e2. doi: 10.2196/med20.2269 (PMC4084770; doi:10.2196/med20.2269)
Supplement: Supplementary file 2 [file med20_v2i2e2_app2.pdf]

Appendix B Overview Table

Authors	Title	Year	Publicatio n	PubMed ID	Message	User	Technolog	Concept	Domain (health plus)	Method words	Stratified Method	over Million	Twitter related Data
Adams A, Lomax G, Santarini A.	Social media & stem cell science: examining the discourse.	2011	Regen Med. 2011 Nov; 6(6 Suppl):121-4.	PMID: 21999274	1	2			stem cell; policy	content analysis	Analytic	tens	35; 50 tweets
Bollen J, Gonçalves B, Ruan G, Mao H.	Happiness is assortative in online social networks.	2011	Artif Life. 2011 Summer; 17(3):237-51. Epub 2011 May 9.	PMID: 21554117	2	1			happiness	graph; mathematical; content analysis	Analytic	millions	6 months of individual users tweets; 129 million tweets; complete history of 4 million users
Bonetta L.	Should you be tweeting?	2009	Cell. 2009 Oct 30; 139(3):452-3.	PMID: 19879830				1	general	review	Examination	none	explaining Twitter
Bristol TJ.	Twitter: consider the possibilities for continuing nursing education.	2010	J Contin Educ Nurs. 2010 May; 41(5):199-200. doi: 10.3928/00220124-20100423-09.	PMID: 20481418				1	education; nursing	review	Examination	none	explore educational use
Bush H.	Time to tweet?	2009	Hosp Health Netw.	PMID:				1	marketing	review	Examination	none	explaining

			2009 Jun; 83(6):46, 48, 51.	19606626									Twitter
Chew C, Eysenbach G.	Pandemics in the age of Twitter: content analysis of Tweets during the 2009 H1N1 outbreak.	2010	PLoS One. 2010 Nov 29; 5(11):e14118.	PMID: 21124761	1				pandemic; flu	content analysis	Analytic	millions	2 million tweets
Collier N, Son NT, Nguyen NM.	OMG U got flu? Analysis of shared health messages for bio-surveillance.	2011	J Biomed Semantics. 2011 Oct 6; 2 Suppl 5:S9.	PMID: 22166368 [PubMed - in process] PMCID: PMC3239309	1				surveillance; flu	Classification; Content analysis; automation; Machine intelligence	Analytic	millions	5283 tweets; from timeline, applied the approach to the now defunct Edinburgh corpus (97 million tweets)
Cuddy C, Graham J, Morton-Owens EG.	Implementing Twitter in a health sciences library.	2010	Med Ref Serv Q. 2010 Oct; 29(4):320-30.	PMID: 21058176				1	libraries	investigation	Examination	none	experience report no data
Dodds PS, Harris KD, Kloumann IM, Bliss CA, Danforth CM.	Temporal patterns of happiness and information in a global social network: hedonometrics and Twitter.	2011	PLoS One. 2011; 6(12):e26752. Epub 2011 Dec 7.	PMID: 22163266 [PubMed - in process] PMCID: PMC3233600	1				happiness	algorithmic; statistical; content analysis; comparative analysis; survey	Analytic	millions	4.6 billions expressions (tweets)

Dörk M, Gruen D, Williamson C, Carpendale S.	A visual backchannel for large-scale events.	2010	IEEE Trans Vis Comput Graph. 2010 Nov-Dec; 16(6):1129-38.	PMID: 20975151	2		1		technical	system implementation	Design and Development	none	technical no data
Eysenbach G.	Can tweets predict citations? Metrics of social impact based on Twitter and correlation with traditional metrics of scientific impact.	2011	J Med Internet Res. 2011 Dec 19; 13(4):e123.	PMID: 22173204 [PubMed - in process] PMCID: PMC3278109	1				publications; citations; impact factor	mined; correlation analysis; classification analysis	Analytic	thousands	4208 tweets
Fox BI, Varadarajan R.	Use of Twitter to encourage interaction in a multi-campus pharmacy management course.	2011	Am J Pharm Educ. 2011 Jun 10; 75(5):88.	PMID: 21829262	1			2	pharmacy; education	survey; experimental	Examination	thousands	1800 tweets; 131 surveys
Franko OI.	Twitter as a communication tool for orthopedic surgery.	2011	Orthopedics. 2011 Nov; 34(11):873-6.	PMID: 22050252		1			orthopedics	survey	Analytic	tens	412 profiles
Golder SA, Macy MW.	Diurnal and seasonal mood vary with work, sleep, and	2011	Science. 2011 Sep 30; 333(6051):1878-81	PMID: 21960633	1				happiness; sleep	Text analysis; statistical	Analytic	millions	millions of tweets across 84 identifiable

	daylength across diverse cultures.												countries; 509 million messages
Gonçalves B, Perra N, Vespignani A.	Modeling users' activity on twitter networks: validation of Dunbar's number.	2011	PLoS One. 2011; 6(8):e22656. Epub 2011 Aug 3.	PMID: 21826200	2	1			Not health; network	analysis; simulation	Analytic	millions	1.7 million individuals; 380 million tweets
González-Bailón S, Borge-Holthoefer J, Rivero A, Moreno Y.	The Dynamics of Protest Recruitment through an Online Network.	2011	Sci Rep. 2011; 1:197. Epub 2011 Dec 15.	PMID: 22355712		1				content analysis; graph	Analytic	thousands	87,569 users and tracks a total of 581,750 protest messages
Heaivilin N, Gerbert B, Page JE, Gibbs JL.	Public health surveillance of dental pain via Twitter.	2011	J Dent Res. 2011 Sep; 90(9):1047-51. Epub 2011 Jul 18.	PMID: 21768306	1				dental	content analysis	Analytic	tens	722 tweets from 4859 collected over 7 days related to dental pain
Kukreja P, Heck Sheehan A, Riggins J.	Use of social media by pharmacy preceptors	2011	Am J Pharm Educ. 2011 Nov 10; 75(9):176.	PMID: 22171104 [PubMed - in process] PMCID: PMC3230337				1	pharmacy preceptors	survey	Examination	tens	315 surveys
McNeil K, Brna PM, Gordon KE.	Epilepsy in the Twitter era: A need to re-tweet the way	2011	Epilepsy Behav. 2012 Feb; 23(2):127-30. Epub 2011 Nov 30.	PMID: 22134096	1				seizure; epilepsy	qualitative; content analysis;	Analytic	thousands	10622 tweets (half excluded)

	we think about seizures.												
Mistry V.	Critical care training: using Twitter as a teaching tool.	2011	Br J Nurs. 2011 Nov 10-23; 20(20):1292-6.	PMID: 22068003	2			1	nursing; education	experimental	Design and Development	tens	Tweets from 2 small groups of students (12 in each group)
Prochaska JJ, Pechmann C, Kim R, Leonhardt JM.	Twitter=quitter? An analysis of Twitter quit smoking social networks.	2011	Tob Control. 2011 Jul 5.	PMID: 21730101		1			smoking	content analysis	Analytic	tens	152 accounts
Qiu L, Leung AK, Ho JH, Yeung QM, Francis KJ, Chua PF.	Understanding the psychological motives behind microblogging.	2010	Stud Health Technol Inform. 2010; 154:140-4.	PMID: 20543286		1				examination	Examination	tens	2 studies, 74 and 70 students
Reips UD, Garaizar P.	Mining twitter: a source for psychological wisdom of the crowds.	2011	Behav Res Methods. 2011 Sep; 43(3):635-42.	PMID: 21701948			1		psychology	system development; text mining	Design and Development	thousands	technology based project placing tweets in location, demoed with 10,000s tweets
Scanfeld D, Scanfeld V, Larson EL.	Dissemination of health information	2010	Am J Infect Control. 2010 Apr; 38(3):182-8.	PMID: 20347636	1				health information; antibiotics	content analysis; mined	Analytic	thousands	1000 tweets from

	through social networks: twitter and antibiotics.												52,000 mentioning key terms
Schneider A, Jackson R, Baum N.	Social media networking: Facebook and Twitter.	2010	J Med Pract Manage. 2010 Nov-Dec; 26(3):156-7.	PMID: 21243885				1	marketing	review	Examination	none	no data general advice
Signorini A, Segre AM, Polgreen PM.	The use of Twitter to track levels of disease activity and public concern in the U.S. during the influenza A H1N1 pandemic.	2011	PLoS One. 2011 May 4; 6(5):e19467.	PMID: 21573238	1				disease	content analysis; model, statistics	Analytic	millions	approx 1 million and 4 million (from alarger collection)
Stieger S, Burger C.	Let's go formative: continuous student ratings with Web 2.0 application Twitter.	2010	Cyberpsychol Behav Soc Netw. 2010 Apr; 13(2):163-7.	PMID: 20528272				1	Education	system design; evaluation	Design and Development	tens	main study 20 tweeting participant; responding to closed questions on 10 unis (main study)
Su XY, Suominen H, Hanlen L.	Machine intelligence for health information: capturing concepts and	2011	Stud Health Technol Inform. 2011; 168:150-7.	PMID: 21893923			1		Search; health information	System implementation; Machine intelligence; normalisation	Design and Development	thousands	corpus 300,000 tweets

	trends in social media via query expansion.												
Sullivan SJ, Schneiders AG, Cheang CW, Kitto E, Lee H, Redhead J, Ward S, Ahmed OH, McCrory PR.	What's happening?' A content analysis of concussion-related traffic on Twitter.	2011	Br J Sports Med. 2011 Mar 15. [Epub ahead of print]	PMID: 21406451	1				concussion; education	content analysis	Analytic	thousands	3488 tweets identified, 1000 were randomly selected
Takao H, Murayama Y, Ishibashi T, Karagiozov KL, Abe T.	A new support system using a mobile device (smartphone) for diagnostic image display and treatment of stroke.	2011	Stroke. 2012 Jan; 43(1):236-9. Epub 2011	PMID: 21998052			1		stroke; telemedicine	system development	Design and Development	tens	this is a tech project; trialled on 64 possible stroke victims
